# Targeted release of stromal cell-derived factor-1α by reactive oxygen species-sensitive nanoparticles results in bone marrow stromal cell chemotaxis and homing, and repair of vascular injury caused by electrical burns

**DOI:** 10.1371/journal.pone.0194298

**Published:** 2018-03-12

**Authors:** Fang He, Peng-Fei Luo, Tao Tang, Fang Zhang, He Fang, Shi-Zhao Ji, Yu Sun, Guo-Sheng Wu, Bo-Han Pan, Zhi-Bao Huo, Guang-Yi Wang, Zhao-Fan Xia

**Affiliations:** 1 Department of Burn Surgery, the Second Military Medical University affiliated Changhai Hospital, Shanghai, China; 2 Department of Burn Surgery, the Nanjing Medical University affiliated Suzhou Hospital, Jiangsu, China; 3 Department of Surgery, Navy Hospital of PLA, Shanghai, China; 4 School of Environmental Science and Engineering, Shanghai Jiao Tong University, Shanghai, China; Ann and Robert H Lurie Children's Hospital of Chicago, Northwestern University, UNITED STATES

## Abstract

Rapid repair of vascular injury is an important prognostic factor for electrical burns. This repair is achieved mainly via stromal cell-derived factor (SDF)-1α promoting the mobilization, chemotaxis, homing, and targeted differentiation of bone marrow mesenchymal stem cells (BMSCs) into endothelial cells. Forming a concentration gradient from the site of local damage in the circulation is essential to the role of SDF-1α. In a previous study, we developed reactive oxygen species (ROS)-sensitive PPADT nanoparticles containing SDF-1α that could degrade in response to high concentration of ROS in tissue lesions, achieving the goal of targeted SDF-1α release. In the current study, a rat vascular injury model of electrical burns was used to evaluate the effects of targeted release of SDF-1α using PPADT nanoparticles on the chemotaxis of BMSCs and the repair of vascular injury. Continuous exposure to 220 V for 6 s could damage rat vascular endothelial cells, strip off the inner layer, significantly elevate the local level of ROS, and decrease the level of SDF-1α. After injection of Cy5-labeled SDF-1α-PPADT nanoparticles, the distribution of Cy5 fluorescence suggested that SDF-1α was distributed primarily at the injury site, and the local SDF-1α levels increased significantly. Seven days after injury with nanoparticles injection, aggregation of exogenous green fluorescent protein-labeled BMSCs at the injury site was observed. Ten days after injury, the endothelial cell arrangement was better organized and continuous, with relatively intact vascular morphology and more blood vessels. These results showed that SDF-1α-PPADT nanoparticles targeted the SDF-1α release at the site of injury, directing BMSC chemotaxis and homing, thereby promoting vascular repair in response to electrical burns.

## Introduction

Electrical burns are unique accidents in modern industrial society. Although their incidence is not high, the damage is three dimensional with sandwich-like and progressive necrosis characteristics, resulting in serious damage in local tissues. The amputation rate in hospitalized patients exceeds 30%, and retained limbs are often associated with varying degrees of dysfunction [1, 2]. Blood is a good conductor of electricity. After an electric current enters the body, it passes through blood causing blood vessel damage. Tissue ischemic and hypoxic injury resulting from vascular damage is an important reason for the progressive necrosis of electrical burns. At present, there is no clinical treatment effective for reducing vascular injury or promoting vascular repair. Therefore, how to induce the rapid repair of local blood vessels caused by electrical burns is important in reducing tissue necrosis and improving the prognosis.

Bone marrow stromal cells (BMSCs) have the potential to develop into multiple cell types [3]. Under the pathological conditions of burns, BMSCs can quickly mobilize from the bone marrow into the peripheral circulation, translocate to the injury site, and differentiate into endothelial progenitor cells (EPCs) that further differentiate into vascular endothelial cells to repair the injury [4, 5]. The mobilization and aggregation of BMSCs depends primarily on chemotaxis induced by stromal cell-derived factor (SDF)-1α [6, 7]. The effect of SDF-1α on BMSCs chemotaxis relies on a local high concentration, as well as the SDF-1α gradient in the circulation. Therefore, establishment of these parameters is essential for chemotaxis and the capture of stem cells for vascular repair.

Formation of local high concentrations of SDF-1α at the injury site, and creating an SDF-1α gradient in the circulation by the exogenous administration of this factor, remain a challenge. Direct intravascular injection of SDF-1α results in its rapid dilution in the blood. Thus, local effective concentration at the injury site cannot be established continuously. Direct injection of SDF-1α in the tissue may result in its uneven distribution and rapid degradation. Therefore, its efficiency in entering the circulation by this route is uncertain. With the development of drug carrier materials, SDF-1α can be delivered in a biodegradable nanoparticle system. This not only effectively prevents its rapid degradation in the body, but also targets SDF-1α to the effective sites in the body, achieving the goal of directional and long-term release [8–10].

To achieve the targeted release of drugs carried by nanoparticles, it is essential to determine the specific physical, chemical, and biological characteristics at the lesion site. Reactive oxygen species (ROS) are pathogenic factors within in an organism [11], but can be utilized for the targeted release of nanoparticle-carried drugs. In our previous study, the ROS-sensitive thioketal polymer, PPADT, was used as a nanoparticle carrier to encapsulate SDF-1α, forming SDF-1α-PPADT nanoparticles [12]. These nanoparticles degrade and release drugs in response to high ROS concentrations in tissue lesions, achieving the goal of targeted therapy. The current study used a rat vascular injury model of electrical burns. SDF-1α-PPADT nanoparticles were injected through the tail vein to evaluate the effects of targeted release of SDF-1α, directional chemotaxis of BMSCs, and repair of vascular injury caused by electrical burns.

## Materials and methods

### Experimental animals

Male adult Sprague Dawley rats (200–250g) and six-week-old male nude mice (20–22g) were purchased from the Experimental Animal Center of the Second Military Medical University (Shanghai, China). Animals were housed in cages under controlled conditions (23±3°C, 50±5% humidity, 12 h day/night rhythm) with ad libitum food and water. The cages were swept and the absorbent bedding materials were replaced every day. Animals were anaesthetized with an intraperitoneal injection of 40 mg/kg sodium pentobarbital (1%). Immediately after the electrical burns, an appropriate amount of silver sulfadiazine cream was applied to the injury site. Buprenorphine (0.02 mg/kg) was administered through tail vein 6 h and 18 h after the electrical burns to minimized animal suffering and distress. During the study, animals were monitored at least three times daily. The food intake, water intake and body weight of animals in a week were paid attention to assess animal health. Animals were euthanized by an overdose of sodium pentobarbital (200 mg/kg) when tissues were collected.

This study was carried out in strict accordance with the Guide for Care and Use of Laboratory Animals published by U.S. National Institutes of Health (NIH) (publication No. 96–01). All animal experiments were approved by the Institutional Animal Care and Use Committee of the SMMU.

### Materials and reagents

The equipment used for generating electrical burns was as follows: the adjustable voltage regulator (0-250V; rated power, 2000W), the power supply switch, the microcomputer time-controlled switch (accurate to 1 sec), the current meter (range 0-600mA; accuracy, 1mA), the voltage meter (range 0-450V; accuracy, 10V), two copper electrode clips, and two copper electrode plates (3mm×3mm), were purchased from Delixi Electric (Zhejiang, China). The CD31 antibody and the donkey anti-goat secondary antibody were obtained from Santa Cruz Biotechnology (Santa Cruz, CA, USA). The ROS fluorescence detection kit was purchased from Genmed Pharmaceutical Technology (Shanghai, China). First Strand cDNA Synthesis Kit was purchased from Takara Bio (Dalian, China). PCR primers were synthesized by Sangon Biotech (Shanghai, China). ELISA kit of SDF-1α was obtained from Abnova (Taipei, Taiwan). Cell culture reagents were purchased from Invitrogen (Carlsbad, CA, USA). If not stated otherwise, reagents were purchased from Sigma-Aldrich (St Louis, MO, USA).

### Establishing electrical burn vascular injury model

**Fig 1:**
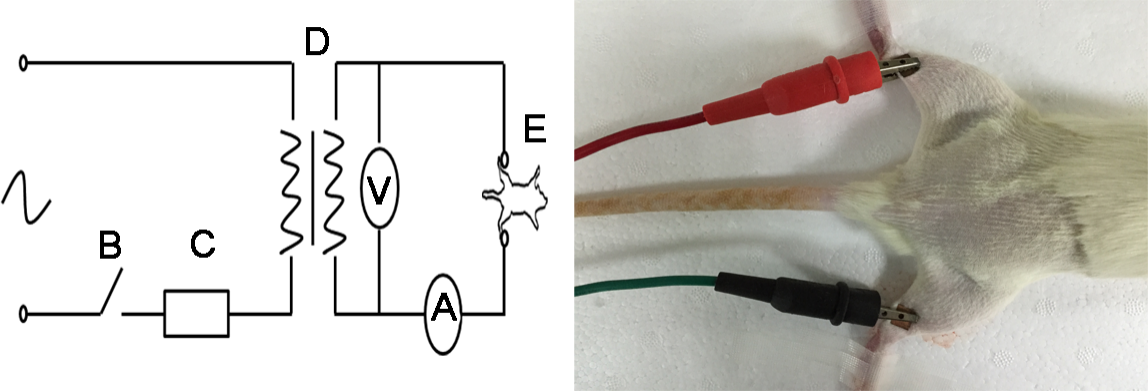
Diagram of the equipment used for generating electrical burns. B: Power supply switch, C: Microcomputer time-controlled switch, D: Voltage regulator, V: Voltage meter, A: Current meter, E: Experimental animal.

The equipment for generating electrical burns was designed and made by our group as described previously (Fig 1) [13]. After anesthetization, the hair on the hip and hind limbs was removed. The rat was then placed in the prone position with limbs extended and fixed to the bench. An appropriate amount of conductive paste was smeared in the region 0.5 cm above the ankle of both hind limbs, and an electrode clip (pressure 5N) was used to fix the electrode plate at this position. The electrode clips were connected to the positive and negative poles of the AC power supply. After completing the electrical circuit, rats were shocked continuously for up to 6 s at 220 V. The maximum current passing through the limbs during the shock was recorded. Rats in the sham electrical burn group were anesthetized and connected to the electrodes the same way as the electrical burn group, but without electrical shock.

Six hours after injury, an overdose of anesthetic was given to euthanize each shocked and control rat. Muscle tissue along the medial extremity, including the main arteries at the site of electrical shock, was collected. Tissues at the same location were collected from the control rats. Samples were fixed with 4% paraformaldehyde, embedded in paraffin, sectioned, and stained to observe the pathological changes of the vascular tissue.

### Identification of vascular injury resulting from electrical burns

A total of 12 rats were divided randomly into two equal groups: 6 s electrical burn and sham. Shock, sample collection, fixation, and slide preparation were performed as described above. In addition to conventional hematoxylin and eosin staining, immunohistochemical staining with the CD31 antibody was performed. Immunohistochemical staining utilized 5μm thick paraffin sections that were deparaffinized and rehydrated. Antigen retrieval was performed at high temperature and pressure. Slides were then immersed in a 3% H_2_O_2_-methanol solution to quench endogenous peroxidases, blocked with 4% bovine serum albumin, and incubated with the CD31 antibody (1:100) at 4˚C overnight. A donkey anti-goat secondary antibody (1:400) was added and incubated at room temperature for 1 h. The 3, 3'-diaminobenzidine substrate was added for visualization. Vascular injury was observed under a microscope.

### Measurement of local ROS levels at the injury site

#### Fluorescence detection of ROS

A total of 12 rats were divided randomly into two equal groups: 6 s electrical burn and sham. After shocking, fresh tissues were frozen rapidly for the preparation of cryosections. ROS were detected following the instructions of a fluorescence detection kit. Immediately after completion of all steps (less than 1 h), slides were placed under a fluorescence microscope to observe the distribution of fluorescence in blood vessels.

#### Antioxidant enzyme mRNA levels by real-time polymerase chain reaction (RT-PCR) analyses

A total of 48 rats were divided randomly into two equal groups: 6 s electrical burn and sham. Six rats from each group were sacrificed 0, 1, 3, and 7 days after the injury for sample collection. RT-PCR analyses were performed to detect the mRNA expression levels of superoxide dismutase (SOD), catalase (CAT), and glutathione peroxidase (GSH-Px). TRIzol reagent was used for total RNA extraction, which was then reverse transcribed into cDNA for the subsequent RT-PCR analyses. The relative quantification method (2^-Δ(ΔCt)^) was used to calculate mRNA expression levels. Glyceraldehyde 3-phosphate dehydrogenase (GAPDH) was used as the internal reference. Primer sequences were: SOD forward 5'-CGGCTTCTGTCGTCTCCT-3', reverse 5'-GTTCACCGCTTGCCTTCT-3'; CAT forward 5'-CCTATTGCCGTCCGATTC-3', reverse 5'-AGGGTCCTTCAGGTGAGTTT-3'; GSH-Px forward 5'-CTGGTGGTGCTCGGTTTC-3', reverse 5'-GTGGGATCGTCACTGGGT-3'; GAPDH forward 5'-ACGGCAAGTTCAACGGCACAG-3', reverse 5'- CCACGACATACTCAGCACCAGC -3'.

### Measurement of local SDF-1α levels in injured tissues

A total of 96 rats were divided randomly into four equal groups: sham, and 1, 3, and 6 s electrical burns. At 0, 1, 3, and 7 days after injury, six rats in each group were sacrificed to collect samples. Tissue homogenates were prepared and centrifuged at 10,000 ×g at 4˚C for 20 min to collect supernatants. SDF-1α concentrations in the supernatants were determined according to the instructions of the SDF-1α ELISA kit.

### SDF-1α-PPADT nanoparticle preparation and verification of ROS-sensitive targeted release of SDF-1α

Nanoparticles were prepared according to the procedures described in our previous study [12]. ROS-sensitive PPADT was synthesized, and then nanoparticles containing the SDF-1α protein were generated using the multiple emulsion solvent evaporation method. The size of the nanoparticles was 110 nm, and the drug loading capacity was 1.8%.

The SDF-1α protein was labeled with Cy5 fluorescent dye and Cy5-SDF-1α-PPADT nanoparticles were prepared according to the method described above. Nine nude mice were divided randomly into three equal groups. Two seconds × 220 V was used for making electrical burns in mice using the same equipment with 3mm×3mm electrode plates fixed at 0.2 cm above the ankle of both hind limbs, other procedures followed the same protocol described above in rats. Group A was the sham electrical burn group. Groups B and C were the electrical burn groups. After 12 h, tail vein injections were performed: group A received 10mg Cy5-SDF-1α-PPADT nanoparticles, group B, 0.18mg Cy5-SDF-1α, and group C, 10mg Cy5-SDF-1α-PPADT nanoparticles. Twelve hours after the injection, an in vivo imaging system (IVIS LaminqII, Caliper LifeSciences, Alameda, CA, USA) was used to observe the distribution of Cy5 fluorescence at the excitation wavelength of 649 nm.

A total of 120 rats were divided randomly into four equal groups: sham + 18 μg/kg SDF-1α; sham + 1mg/kg SDF-1α-PPADT nanoparticles; 6 s electrical burn + 18 μg/kg SDF-1α; and 6 s electrical burn + 1mg/kg SDF-1α-PPADT nanoparticles. At 0, 6, 12, 24, and 48 h, six rats from each group were sacrificed for sample collection. The SDF-1α concentration in local tissue at the injury site was determined using ELISA.

### Directional chemotaxis and homing of BMSCs

Green fluorescent protein (GFP)-labeled BMSCs (Catalog No. RASMX-01101) were purchased from Cyagen Biosciences (Guangzhou, China). After continuous shock for 6 s, 18 rats were divided randomly into three equal groups. Tail vein injection was performed once a day as follows: A) 5 × 10^6^ GFP-BMSCs; B) 5 × 10^6^ GFP-BMSCs + 18 μg/kg SDF-1α; and C) 5 × 10^6^ GFP-BMSCs + 1mg/kg SDF-1α-PPADT nanoparticles. Samples were collected 7 days after injury for cryosection preparation. CD31 immunofluorescence staining was performed, and the numbers of GFP+ and CD31+ cells were detected under a fluorescence microscope.

### Effects of SDF-1α-PPADT nanoparticles on vascular repair

After continuous shock for 6 s, 18 rats were divided randomly into three equal groups. Tail vein injection was performed once a day as follows: A) 0.1 mL normal saline; B) 18 μg/kg SDF-1α; and C) 1 mg/kg SDF-1α-PPADT nanoparticles. Ten days after the injury, rats were sacrificed for sample collection. Samples were fixed with 4% paraformaldehyde, embedded in paraffin, and sectioned. This was followed by hematoxylin and eosin, and CD31 immunohistochemical staining. Pathological changes of the vascular tissues were observed under a microscope.

### Statistical analysis

All data were processed using SPSS 23.0 software (IBM, Chicago, IL, USA) and are presented as means ± SEM. Data were assessed using an analysis of variance and the Bonferroni post-hoc test. A p value less than 0.05 was considered statistically significant.

## Results

### General observations after the electrical burn

All animals survived after electrical burns. The maximum current passing through the limbs during electrical shock was 180 ± 36 mA. A third-degree burn wound formed in the area where the electrode plate was applied on rats and mice. Rats and mice were limping on both hind limbs after electrical burns.

### Electrical burns caused vascular damage

**Fig 2:**
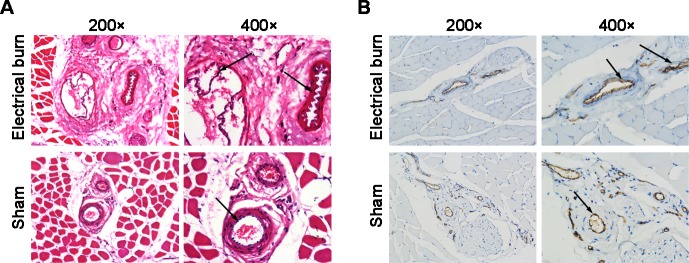
Vascular injury caused by 6 s electrical burns in rats. (A) Hematoxylin and eosin staining of muscle and vascular tissues. In the electrical burn group, vascular endothelial cells form protrusions into the lumen; some endothelial cells are stripped from the lumen and thrombi appear in parts of the blood vessels (arrow). In the sham electrical burn group, endothelial cells are intact and organized (arrow). Images on the left are 200 × magnification. Images on the right are 400 × magnification. (B) CD31 immunohistochemical staining of muscle and vascular tissues. CD31 staining is brown. The nuclei are counterstained in blue. The inner layer of CD31+ blood vessels is detached and the lumens are narrow in the electrical burn compared to the sham group (arrow). Images on the left are 200 × magnification. Images on the right are 400 × magnification.

Hematoxylin and eosin staining showed that, in the 6 s electrical burn group, vascular endothelial cells formed protrusions into the lumen and were discontinuous. Some endothelial cells were even stripped from the lumen, and thrombi formed in some parts of the blood vessels (Fig 2A, first row, arrow). In the sham electrical burn group, vascular endothelial cells were smooth, closely connected, and organized (Fig 2A, second row, arrow). Immunohistochemical staining showed that, compared to the sham group, CD31+ vessels in the electrical burn group exhibited a dissociated inner layer and lumen narrowing (Fig 2B, arrow). These results suggested that continuous shock for 6 s at 220 V resulted in significant vascular damage in rats.

### Local ROS levels were increased significantly at the injury site

**Fig 3:**
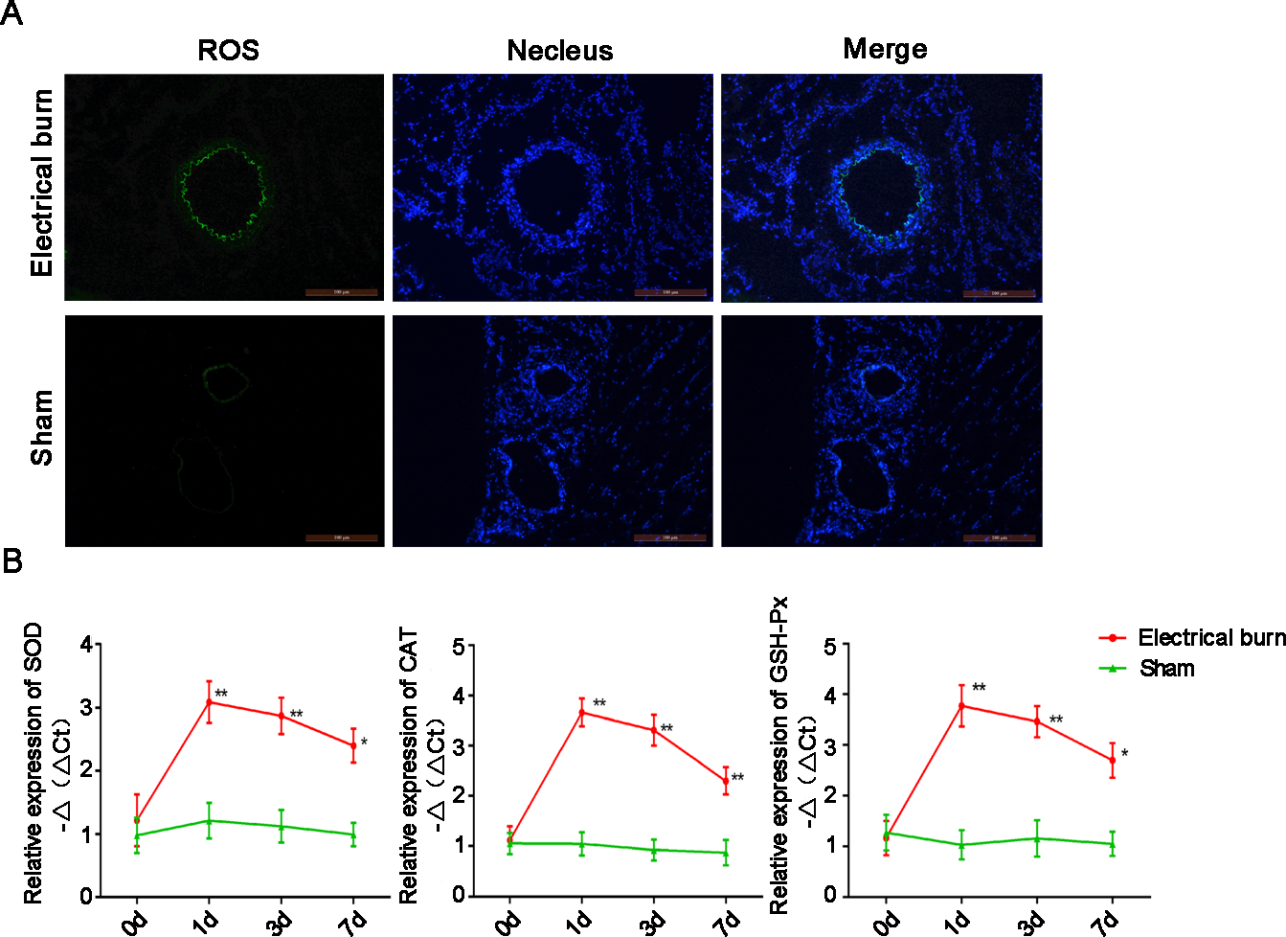
Reactive oxygen species (ROS) levels are increased significantly at the injury site. (A) Fluorescence staining for ROS in muscle and vascular tissue sections. Green fluorescence indicates ROS and blue fluorescence indicates the 4', 6-diamidino-2-phenylindole-stained nuclei. Bar, 100 μm. (B) mRNA levels of antioxidant enzymes increase locally at the injury site. mRNA levels of SOD, CAT, and GSH-Px were detected using the real-time polymerase chain reaction. Data are presented as means ± SEM (n = 6). *p < 0.05, **p < 0.01 versus the sham electrical burn group.

Fluorescence detection of ROS showed that, in the 6 s electrical burn group, green fluorescence in the blood vessels was obvious, whereas in the sham electrical burn group green fluorescence was weak or even absent (Fig 3A). These results suggested that the local ROS levels in vascular tissue of rats injured by electrical burns were significantly elevated compared to that in normal tissue.

Antioxidant enzymes use redox reactions to convert peroxides into substances of lower or no toxicity. To determine whether ROS levels were increased locally at the injury site, RT-PCR analyses were performed to detect the mRNA expression levels of SOD, CAT, and GSH-Px in rats. The results showed that on days 1, 3, and 7 after the 6 s electrical burn injury, SOD mRNA levels were 3.78, 3.45, and 2.79 times higher, respectively, than those in the sham group (Fig 3B). CAT mRNA levels were 6.28, 5.61, and 2.72 times higher than those in the sham electrical burn group on days 1, 3, and 7, respectively (Fig 3B). GSH-Px mRNA levels were 7.26, 4.73, and 3.39 times higher than those in the sham group on days 1, 3, and 7, respectively (p < 0.05) (Fig 3B). These results suggested that local ROS levels in blood vessel tissues damaged by electrical burns were higher than those in normal tissue.

### Within a certain range, the extent of local electrical burn injury was inversely related to the SDF-1α level

**Fig 4:**
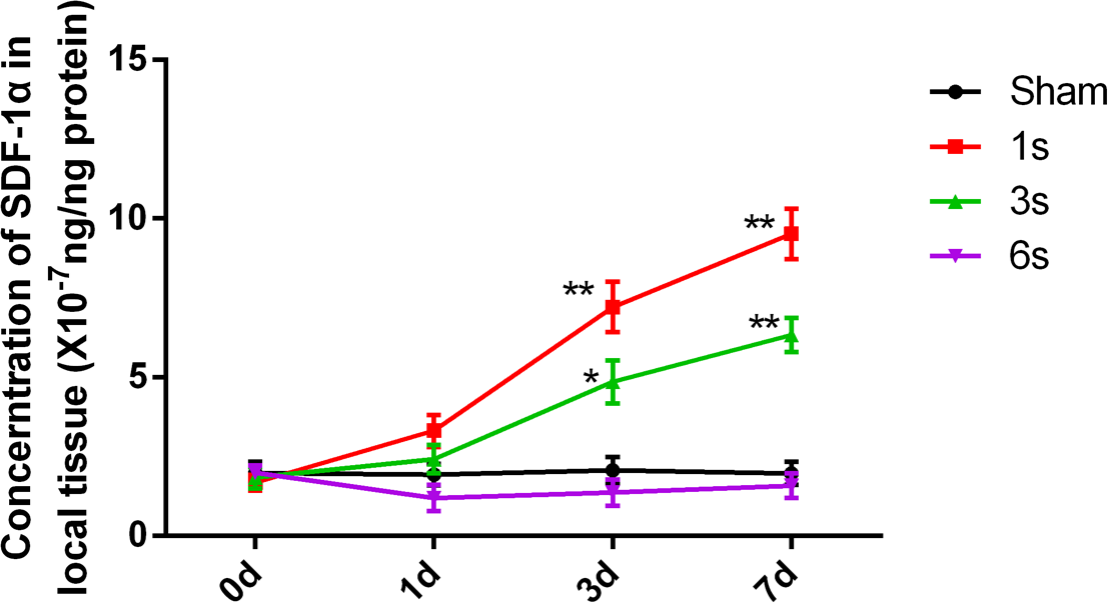
The severity of local electrical burns is inversely related to SDF-1α levels within a certain range of injury. Samples were collected at different time points after different shock durations. Tissue SDF-1α concentrations were determined using an ELISA. Data are presented as means ± SEM (n = 6), *p < 0.05, **p < 0.01 versus the sham electrical burn group.

To determine the relationship between local tissue SDF-1α concentrations and the extent of injury, shock time was adjusted to produce different degrees of injury in rats. SDF-1α concentrations were then measured using the ELISA method. SDF-1α concentrations following 1 and 3 s electrical shocks on days 3 and 7 after the injury were significantly higher than those of the sham electrical burn group. However, SDF-1α concentrations in the 6 s electrical shock group on days 1, 3, and 7 after the injury were lower and not significantly different from the sham group (Fig 4). These results indicated that mild electrical burns increased local SDF-1α production, and with increasing severity of the burns, SDF-1α synthesis was reduced.

### SDF-1α-PPADT nanoparticles had good targeted drug release properties

**Fig 5:**
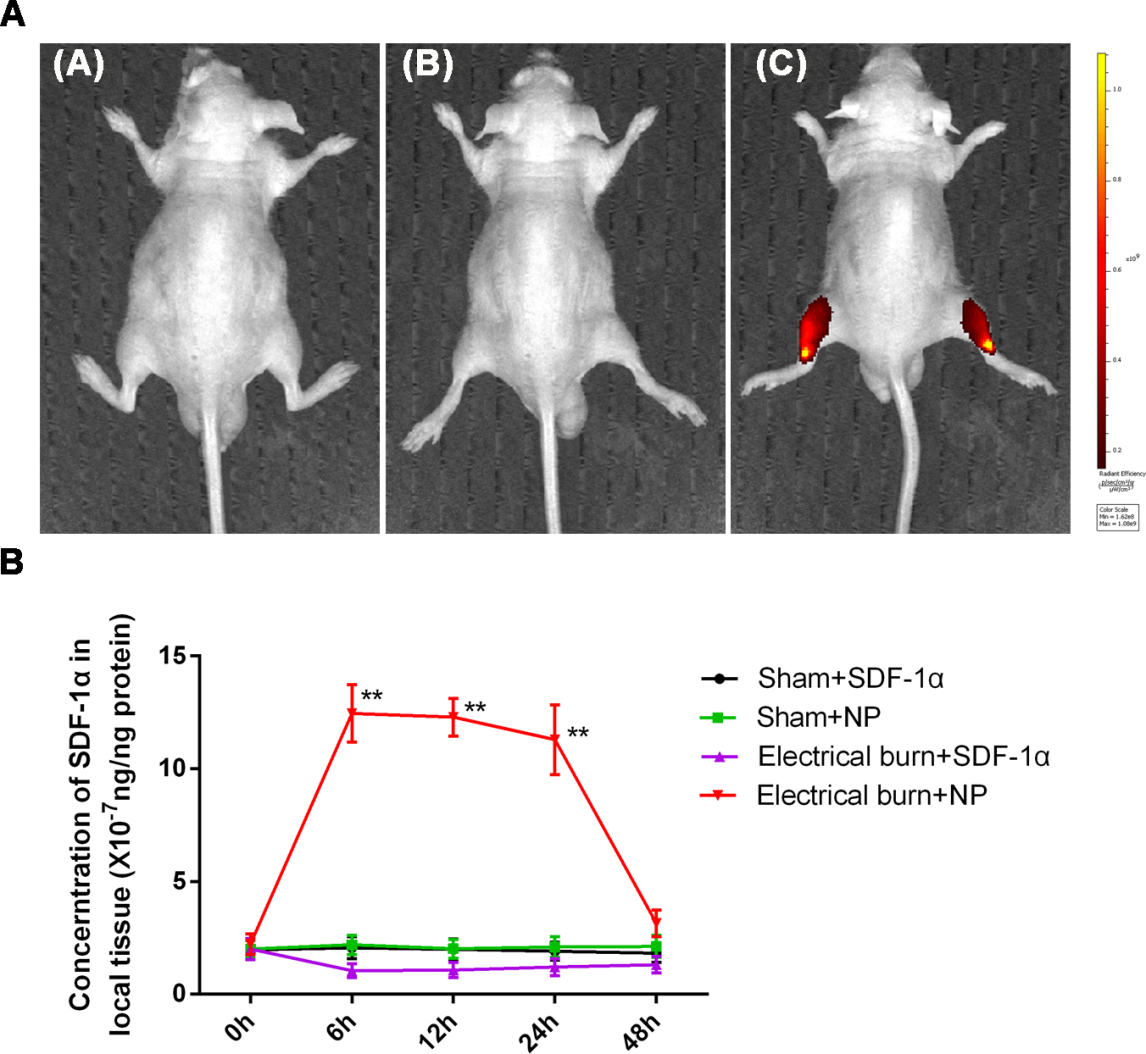
SDF-1α-PPADT nanoparticles have targeted drug release properties in mice. (A) Group A is the sham electrical burn group. Groups B and C were the electrical burn groups. After 12 h, tail vein injections were performed: group A, 10 mg Cy5-SDF-1α-PPADT nanoparticles; group B, 0.18 mg Cy5-SDF-1α; and group C, 10 mg Cy5-SDF-1α-PPADT nanoparticles. Twelve hours after injection, the distribution of Cy5 fluorescence was observed using an *in vivo* imaging system. (B) Samples were collected from each group at different time points after the injury. Local tissue SDF-1α concentrations were determined using an ELISA. NP is SDF-1α-PPADT nanoparticles. Data are presented as means ± SEM (n = 6), **p < 0.01 versus the other three control groups.

To determine whether SDF-1α-PPADT nanoparticles could target SDF-1α release in the injured tissue, distribution and concentrations of SDF-1α protein were assessed. SDF-1α labeled with the Cy5 fluorescent dye was used to prepare Cy5-SDF-1α-PPADT nanoparticles. These particles were injected into mice through the tail vein for in vivo imaging analysis. In nude mice receiving a 2 s electrical burn and 10 mg Cy5-SDF-1α-PPADT nanoparticles, the injury site and surrounding tissues showed obvious fluorescence, while no obvious fluorescence was detected in other parts of the body. In the two control groups (sham burn + 10 mg Cy5-SDF-1α-PPADT nanoparticles and 2 s burn + 0.18 mg Cy5-SDF-1α), no fluorescence was observed (Fig 5A).

The concentration of SDF-1α in local tissue after administration of SDF-1α-PPADT nanoparticles was measured by ELISA. Compared with the other three control groups, electrical burn rats injected with SDF-1α-PPADT nanoparticles showed a significant increase in the concentration of SDF-1α at the electrical burn injury site (Fig 5B). These results indicated that SDF-1α-PPADT nanoparticles could target SDF-1α release to the site of injury, thereby increasing the local SDF-1α concentration.

### SDF-1α-PPADT nanoparticles directed BMSC chemotaxis and homing

**Fig 6:**
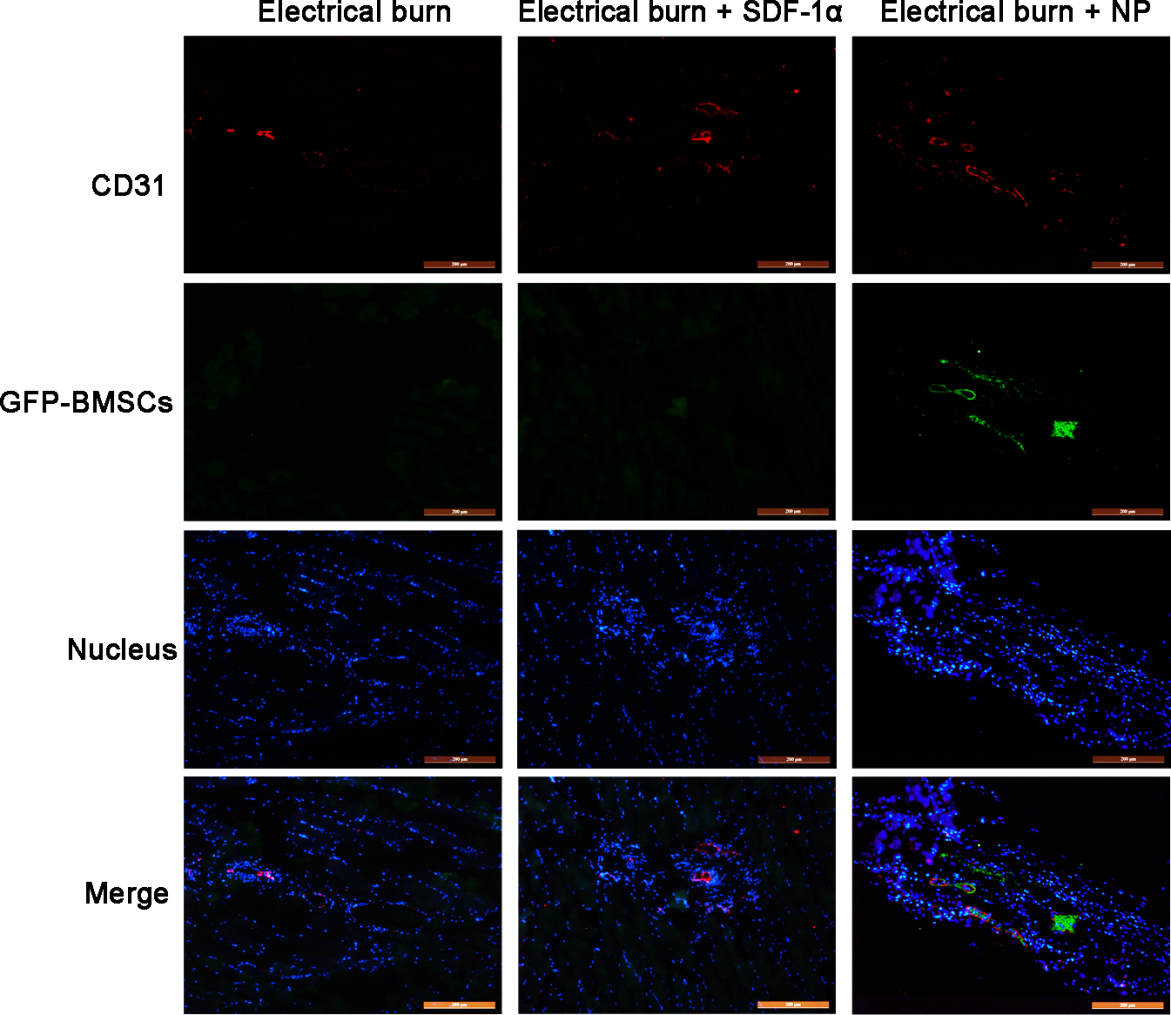
SDF-1α-PPADT nanoparticles direct BMSC chemotaxis and homing in rats. All rats received continuous shock for 6 s and were divided randomly into three equal groups of six. Tail vein injections were performed once a day: (A) 5 × 10^6^ GFP-BMSCs; (B) 5 × 10^6^ GFP-BMSCs + 18 μg/kg SDF-1α; and (C) 5 × 10^6^ GFP-BMSCs + 1 mg/kg SDF-1α-PPADT nanoparticles. Seven days after the injury, samples were collected for cryosection preparation and CD31 immunofluorescence staining. Red fluorescence indicates CD31-labeled vascular endothelial cells. Green fluorescence indicates GFP-labeled BMSCs. Blue fluorescence indicates 4', 6-diamidino-2-phenylindole-stained nuclei. Bar, 200 μm. NP, SDF-1α-PPADT nanoparticles.

To determine whether SDF-1α-PPADT nanoparticles could direct chemotaxis of BMSCs, we concomitantly injected SDF-1α-PPADT nanoparticles and GFP-labeled BMSCs intravenously. Seven days later, pathological analysis showed that GFP-BMSCs clearly accumulated at the injury site in the group injected with SDF-1α-PPADT nanoparticles. In the group treated with only electrical burns, and the group injected with SDF-1α, GFP-BMSCs were not detected (Fig 6). These results suggested that SDF-1α-PPADT nanoparticles can promote BMSCs homing to the injury site, indicative of directed chemotaxis.

### SDF-1α-PPADT nanoparticles promoted vascular repair

**Fig 7:**
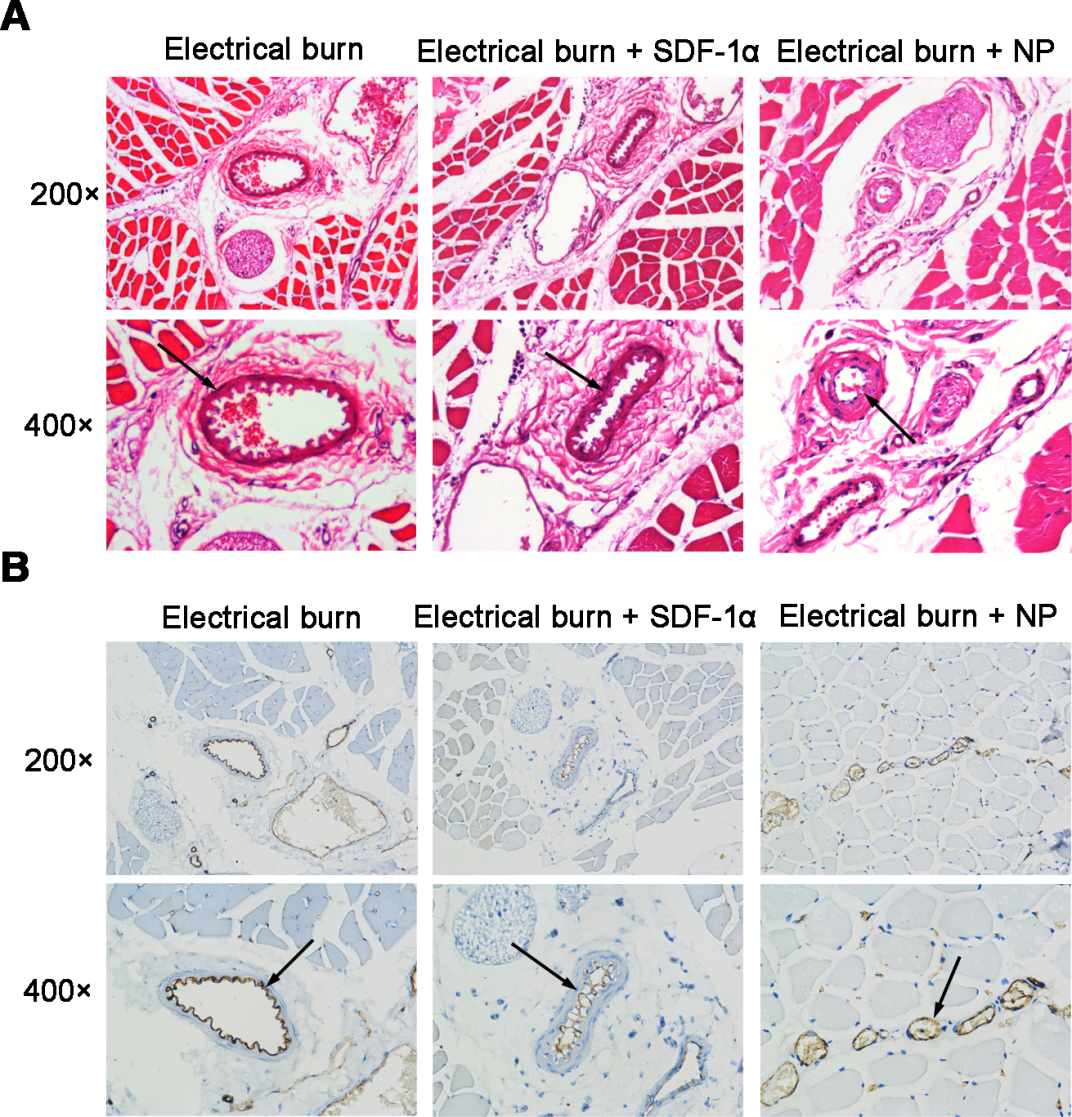
SDF-1α-PPADT nanoparticles promote the repair of electrical burn vascular injury in rats. (A) Hematoxylin and eosin staining of muscle and vascular tissues. Vascular morphology of the 6 s electrical burn group injected with SDF-1α-PPADT nanoparticles is relatively intact. Endothelial cells are better organized and continuous (arrow). In the group treated with only electrical burn and the group injected with SDF-1α, the inner vascular layer formed protrusions into the lumen (arrow). Images on the first rows, 200 × magnification. Images on the second rows, 400 × magnification. (B) CD31 immunohistochemical staining of muscle and vascular tissues. CD31 staining is brown and nucleus staining is blue. In the electrical burn group injected with SDF-1α-PPADT nanoparticles, more CD31+ blood vessels are present, and the lumens are round or oval (arrow). In the group treated with only electrical burn and the group injected with SDF-1α, the inner layer of the blood vessel is detached and the lumens are occluded (arrow). Images on the first rows, 200 × magnification. Images on the second rows, 400 × magnification.

Hematoxylin and eosin staining showed relatively intact vascular morphology in the electrical burn group injected with SDF-1α-PPADT nanoparticles. In addition, the endothelial cell arrangement was better organized and continuous. In the group treated with only electrical burns, and the group injected with SDF-1α, vascular endothelial cells formed protrusions into the lumen (Fig 7A, arrow). Immunohistochemical staining showed that there were more CD31+ blood vessels in the electrical burn group injected with SDF-1α-PPADT nanoparticles, and the lumens were round or oval. However, in the group treated with only simple electrical burns, and the group injected with SDF-1α, the inner layer of the blood vessels was detached, and parts of the lumen were occluded (Fig 7B, arrow). These results suggested that injection of SDF-1α-PPADT nanoparticles promoted vascular repair caused by electrical burns.

## Discussion

Blood vessel injury and repair are important prognostic factors for electrical burns. The latter is achieved via BMSCs homing to the injury site and up regulating the expression of angiogenic factors through paracrine actions such as VEGF, FGF, angiopoietin, HGF. Under the action of these cytokines, BMSCs are differentiated into vascular endothelial cells and form vascular-like structures [3, 4]. The SDF-1α receptor, CXCR4, is highly expressed on the surface of BMSCs and, under various pathological conditions, damaged tissues can release chemokines, SDF-1α, that bind specifically to CXCR4. Secreted SDF-1α reaches the bone marrow through the circulation, which in turn activates matrix metalloproteinase-9 (MMP-9) resulting in the release of soluble Kit-ligand (sKitL), promoting the mobilization of BMSCs into the peripheral circulation. Under the regulation of multiple factors, BMSCs migrate in response to chemotaxis, localizing to the injury site and undergoing directional differentiation, hence participating in repairing the injury [14, 15].

SDF-1α is expressed in dermis, dermal papilla, blood vessels, neurons, and other tissues [16]. SDF-1α is a potent chemotactic agent for BMSCs homing to the injury site [17]. Local high expression of SDF-1α can accelerate tissue vascularization, promoting vascular regeneration [18, 19]. In addition, through autocrine/paracrine effects, SDF-1α can inhibit apoptosis and promote proliferation of stem cells originating in the bone marrow [20, 21]. On the other hand, a lack of SDF-1α in local tissue results in impaired injury repair.

Regionally low expression of SDF-1α is the main reason for the difficult healing of diabetic ulcers, and in vivo tracing shows that local EPC homing is disrupted [22]. The experimental results discussed above suggested that local high concentrations of SDF-1α, and the SDF-1α gradient in the circulation, resulted in chemotaxis, homing, and directional differentiation of BMSCs, which is essential for vascular repair. However, within a certain range, the increasing degree of vascular injury and tissue necrosis caused by electrical burns leads to decreases in the local SDF-1α concentration (Fig 4). This might be the result of severe electrical burns causing extensive tissue damage and necrosis, leaving too little residual surviving tissue to synthesize and secrete a sufficient amount of SDF-1α for repair. In clinical practice, patients with severe electrical burn injury often suffer from progressively worsening vascular damage, which may be related to insufficient local SDF-1α production and impaired repair. Therefore, supplementation with exogenous SDF-1α to enhance BMSC chemotaxis and homing may be essential to promote vascular repair.

Generating a local continuous high concentration of SDF-1α is challenging. Protein and peptide drugs have disadvantages such as poor stability, easy degradation, and a short half-life in the circulation [23]. Direct intravascular injection of SDF-1α results in its rapid dilution in the blood, preventing the formation of an effective concentration gradient. Direct injection of SDF-1α into tissues results in its uneven distribution and easy degradation, and the efficiency of SDF-1α in entering the circulation is uncertain. In our previous study, ROS-sensitive PPADT was used as a nanoparticle carrier to encapsulate SDF-1α, forming SDF-1α-PPADT nanoparticles. Importantly, high concentrations of ROS in the lesions triggered structural changes of PPADT, releasing SDF-1α and achieving targeted therapy. This provided an effective method for achieving high local SDF-1α concentrations at the injury site and yielded a long half-life in the circulation.

There is no universally recognized animal model for studies of vascular injury caused by electrical burns. We used the method of Wan et al. [13] to make the equipment for generating electrical burns. The application of a continuous shock for 6 s at 220 V in rats produced relatively consistent vascular injury. The main blood vessels in rat hind limbs are superficial and their anatomical positions are constant. Therefore, the path for the electrical current was relatively fixed. Histopathological analysis of the damaged tissues showed that the limb vascular injury generated in this model was highly stable, hence suitable for experimental studies of vascular injury and repair resulting from electrical burns (Fig 2).

The key to achieving the targeted release of drugs carried by nanoparticles is to determine specific local physical, chemical, or biological characteristics at the injury site. Oxidative stress is an important mechanism involved in many kinds of injuries [24–26]. Several studies have reported that oxidative stress is increased locally at the sites of vascular injury [27–33] while ROS levels remain low in normal tissues or cells [34, 35]. This study showed that ROS levels in the damaged blood vessels remained high for a long period of time during the course of electrical burns (Fig 3). Therefore, ROS can be used as a specific target for distinguishing damaged from normal tissue.

PPADT is ROS-sensitive due to its thioketal linkages. At high ROS concentrations, these linkages break and PPADT depolymerizes to release the encapsulated drug, thereby achieving targeted release [36]. At present, there are few reports on the application of PPADT as a drug carrier. Wilson et al. used PPADT to package siRNA which was then administered orally to treat ulcerative colitis [37]. Our research used SDF-1α-PPADT nanoparticles to mimic the process of chemotaxis in the circulation to treat vascular disease. SDF-1α was labeled with Cy5 and the nanoparticles were injected intravenously into nude mice. In vivo imaging showed that fluorescence was mainly distributed at the injury site, while no obvious fluorescence was detected in normal tissues (Fig 5A). In addition, we examined the local SDF-1α concentration at the injury site in rats after nanoparticle injection. Local SDF-1α levels were improved significantly by the injection of nanoparticles (Fig 5B). These results demonstrated that SDF-1α-PPADT nanoparticles have good targeted drug release properties.

As mentioned earlier, vascular injury requires the presence of local high concentrations of SDF-1α for BMSC chemotaxis, homing, and differentiation into EPCs, which further differentiate into vascular endothelial cells to achieve repair. To examine whether the repair achieved with SDF-1α-PPADT nanoparticles involved BMSC chemotaxis and homing, GFP-transfected BMSCs were injected intravenously simultaneously for tracing. The results showed that the rats treated with SDF-1α-PPADT nanoparticles had GFP-BMSCs gathering at the site of vascular injury (Fig 6). This confirmed that SDF-1α-PPADT nanoparticles induced BMSC chemotaxis and homing to the injury site. Furthermore, in the rat model with daily tail vein injections of nanoparticles, histopathological analyses of sample sections showed that blood vessel morphology was relatively intact, the endothelial cell arrangement was better organized and continuous, and the number of blood vessels was higher in the nanoparticle treatment than that in the control group (Fig 7). These morphological analyses confirmed that nanoparticles can promote the repair of blood vessel injury.

Overall, this study achieved the targeted release of SDF-1α at the site of electrical burn injury using SDF-1α-PPADT nanoparticles. These particles induced BMSC chemotaxis and homing, and promoted the repair of vascular injury caused by electrical burns. This provides an effective treatment method for vascular repair caused by electrical burns, as well as new ideas and methods for studying the repair of other vascular lesions.

## Supporting information

S1 Dataset The primary data for local ROS levels at the injury site. (XLSX)

S2 Dataset The primary data for local SDF-1α levels in injured tissues. (XLSX)

S3 Dataset The primary data for verification of ROS-sensitive targeted release of SDF-1α. (XLSX)

## References

[1] Pannucci CJ, Osborne NH, Jaber RM, Cederna PS, Wahl WL. Early fasciotomy in electrically injured patients as a marker for injury severity and deep venous thrombosis risk: an analysis of the National Burn Repository. J Burn Care Res. 2010; 31: 882–887. doi: 10.1097/BCR.0b013e3181f93597 20861746 10.1097/BCR.0b013e3181f93597 PMC2976802

[2] Handschin AE, Vetter S, Jung FJ, Guggenheim M, Künzi W, Giovanoli P. A case-matched controlled study on high-voltage electrical injuries vs thermal burns. J Burn Care Res. 2009; 30: 400–407. doi: 10.1097/BCR.0b013e3181a289a6 19349896 10.1097/BCR.0b013e3181a289a6

[3] Bronckaers A, Hilkens P, Martens W, Gervois P, Ratajczak J, Struys T, et al Mesenchymal stem/stromal cells as a pharmacological and therapeutic approach to accelerate angiogenesis. Pharmacol Ther. 2014; 143: 181–196. doi: 10.1016/j.pharmthera.2014.02.013 24594234 10.1016/j.pharmthera.2014.02.013

[4] Allers C, Sierralta WD, Neubauer S, Rivera F, Minguell JJ, Conget PA. Dynamic of distribution of human bone marrow-derived mesenchymal stem cells after transplantation into adult unconditioned mice. Transplantation. 2004; 78: 503–508. 15446307 10.1097/01.tp.0000128334.93343.b3

[5] Foresta C, Schipilliti M, De Toni L, Magagna S, Lancerotto L, Azzena B, et al Blood levels, apoptosis, and homing of the endothelial progenitor cells after skin burns and escharectomy. J Trauma. 2011; 70: 459–465. doi: 10.1097/TA.0b013e3181fcf83c 21307748 10.1097/TA.0b013e3181fcf83c

[6] Ma J, Ge J, Zhang S, Sun A, Shen J, Chen L, et al Time course of myocardial stromal cell-derived factor 1 expression and beneficial effects of intravenously administered bone marrow stem cells in rats with experimental myocardial infarction. Basic Res Cardiol. 2005; 100: 217–223. doi: 10.1007/s00395-005-0521-z 15754085 10.1007/s00395-005-0521-z

[7] Schober A, Knarren S, Lietz M, Lin EA, Weber C. Crucial role of stromal cell-derived factor-1alpha in neointima formation after vascular injury in apolipoprotein E-deficient mice. Circulation. 2003; 108: 2491–2497. doi: 10.1161/01.CIR.0000099508.76665.9A 14581398 10.1161/01.CIR.0000099508.76665.9A

[8] Kommareddy S, Amiji M. Biodistribution and pharmacokinetic analysis of long-circulating thiolated gelatin nanoparticles following systemic administration in breast cancer-bearing mice. J Pharm Sci. 2007; 96: 397–407. doi: 10.1002/jps.20813 17075865 10.1002/jps.20813

[9] Delie F, Blanco-Príeto MJ. Polymeric particulates to improve oral bioavailability of peptide drugs. Molecules. 2005; 10: 65–80. 18007277 10.3390/10010065 PMC6147556

[10] Okuda T, Kawakami S, Akimoto N, Niidome T, Yamashita F, Hashida M. PEGylated lysine dendrimers for tumor-selective targeting after intravenous injection in tumor-bearing mice. J Control Release. 2006; 116: 330–336. doi: 10.1016/j.jconrel.2006.09.012 17118476 10.1016/j.jconrel.2006.09.012

[11] Mittal M, Siddiqui MR, Tran K, Reddy SP, Malik AB. Reactive oxygen species in inflammation and tissue injury. Antioxid Redox Signal. 2014; 20: 1126–1167. doi: 10.1089/ars.2012.5149 23991888 10.1089/ars.2012.5149 PMC3929010

[12] Tang T, Jiang H, Yu Y, He F, Ji SZ, Liu YY, et al A new method of wound treatment: targeted therapy of skin wounds with reactive oxygen species-responsive nanoparticles containing SDF-1α. Int J Nanomedicine. 2015; 10: 6571–6585. doi: 10.2147/IJN.S88384 26527874 10.2147/IJN.S88384 PMC4621221

[13] Wan L, Ma Z, Zhang Z. Experimental study on vascular injury induced by the electricity. Chinese Journal of Forensic Medicine. 2001; 16(1): 19–22

[14] Heissig B, Hattori K, Dias S, Friedrich M, Ferris B, Hackett NR, et al Recruitment of stem and progenitor cells from the bone marrow niche requires MMP-9 mediated release of kit-ligand. Cell. 2002; 109: 625–637. 12062105 10.1016/s0092-8674(02)00754-7 PMC2826110

[15] Petit I, Jin D, Rafii S. The SDF-1-CXCR4 signaling pathway: a molecular hub modulating neo-angiogenesis. Trends Immunol. 2007; 28: 299–307. doi: 10.1016/j.it.2007.05.007 17560169 10.1016/j.it.2007.05.007 PMC2952492

[16] Avniel S, Arik Z, Maly A, Sagie A, Basst HB, Yahana MD, et al Involvement of the CXCL12/CXCR4 pathway in the recovery of skin following burns. J Invest Dermatol. 2006; 126: 468–476. doi: 10.1038/sj.jid.5700069 16385346 10.1038/sj.jid.5700069

[17] Wynn RF, Hart CA, Corradi-Perini C, O'Neill L, Evans CA, Wraith JE, et al A small proportion of mesenchymal stem cells strongly expresses functionally active CXCR4 receptor capable of promoting migration to bone marrow. Blood. 2004; 104: 2643–2645. doi: 10.1182/blood-2004-02-0526 15251986 10.1182/blood-2004-02-0526

[18] Hiasa K, Ishibashi M, Ohtani K, Inoue S, Zhao Q, Kitamoto S, et al Gene transfer of stromal cell-derived factor-1alpha enhances ischemic vasculogenesis and angiogenesis via vascular endothelial growth factor/endothelial nitric oxide synthase-related pathway: next-generation chemokine therapy for therapeutic neovascularization. Circulation. 2004; 109: 2454–2461. doi: 10.1161/01.CIR.0000128213.96779.61 15148275 10.1161/01.CIR.0000128213.96779.61

[19] Tan Y, Shao H, Eton D, Yang Z, Alonso-Diaz L, Zhang H, et al Stromal cell-derived factor-1 enhances pro-angiogenic effect of granulocyte-colony stimulating factor. Cardiovasc Res. 2007; 73: 823–832. doi: 10.1016/j.cardiores.2006.12.015 17258698 10.1016/j.cardiores.2006.12.015 PMC2243257

[20] Lataillade JJ, Clay D, Bourin P, Hérodin F, Dupuy C, Jasmin C, et al Stromal cell-derived factor 1 regulates primitive hematopoiesis by suppressing apoptosis and by promoting G(0)/G(1) transition in CD34(+) cells: evidence for an autocrine/paracrine mechanism. Blood. 2002; 99: 1117–1129. 11830456 10.1182/blood.v99.4.1117

[21] Liu X, Duan B, Cheng Z, Jia X, Mao L, Fu H, et al SDF-1/CXCR4 axis modulates bone marrow mesenchymal stem cell apoptosis, migration and cytokine secretion. Protein Cell. 2011; 2: 845–854. doi: 10.1007/s13238-011-1097-z 22058039 10.1007/s13238-011-1097-z PMC4875294

[22] Restivo TE, Mace KA, Harken AH, Young DM. Application of the chemokine CXCL12 expression plasmid restores wound healing to near normal in a diabetic mouse model. J Trauma. 2010; 69: 392–398. doi: 10.1097/TA.0b013e3181e772b0 20699749 10.1097/TA.0b013e3181e772b0

[23] Misra P, Lebeche D, Ly H, Schwarzkopf M, Diaz G, Hajjar RJ, et al Quantitation of CXCR4 expression in myocardial infarction using 99mTc-labeled SDF-1alpha. J Nucl Med. 2008; 49: 963–969. doi: 10.2967/jnumed.107.050054 18483105 10.2967/jnumed.107.050054 PMC2712574

[24] Rogobete AF, Sandesc D, Papurica M, Stoicescu ER, Popovici SE, Bratu LM, et al The influence of metabolic imbalances and oxidative stress on the outcome of critically ill polytrauma patients: a review. Burns Trauma. 2017; 5: 8 doi: 10.1186/s41038-017-0073-0 28286784 10.1186/s41038-017-0073-0 PMC5341432

[25] Kimura A, Namekata K, Guo X, Noro T, Harada C, Harada T. Targeting Oxidative Stress for Treatment of Glaucoma and Optic Neuritis. Oxid Med Cell Longev. 2017; 2017: 2817252 doi: 10.1155/2017/2817252 28270908 10.1155/2017/2817252 PMC5320364

[26] Pisoschi AM, Pop A. The role of antioxidants in the chemistry of oxidative stress: A review. Eur J Med Chem. 2015; 97: 55–74. doi: 10.1016/j.ejmech.2015.04.040 25942353 10.1016/j.ejmech.2015.04.040

[27] Gomez C, Martinez L, Mesa A, Duque JC, Escobar LA, Pham SM, et al Oxidative stress induces early-onset apoptosis of vascular smooth muscle cells and neointima formation in response to injury. Biosci Rep. 2015; 35(4). doi: 10.1042/BSR20140122 26182434 10.1042/BSR20140122 PMC4613704

[28] Wang J, Wang S, Li M, Wu D, Liu F, Yang R, et al The Neuropilin-1 Inhibitor, ATWLPPR Peptide, Prevents Experimental Diabetes-Induced Retinal Injury by Preserving Vascular Integrity and Decreasing Oxidative Stress. PLoS One. 2015; 10: e0142571 doi: 10.1371/journal.pone.0142571 26554379 10.1371/journal.pone.0142571 PMC4640834

[29] Burke A, Fitzgerald GA. Oxidative stress and smoking-induced vascular injury. Prog Cardiovasc Dis. 2003; 46: 79–90. 12920701 10.1016/s0033-0620(03)00076-8

[30] Yamada T, Egashira N, Imuta M, Yano T, Yamauchi Y, Watanabe H, et al Role of oxidative stress in vinorelbine-induced vascular endothelial cell injury. Free Radic Biol Med. 2010; 48: 120–127. doi: 10.1016/j.freeradbiomed.2009.10.032 19837156 10.1016/j.freeradbiomed.2009.10.032

[31] Xu Y, Zhu J, Hu X, Wang C, Lu D, Gong C, et al CLIC1 Inhibition Attenuates Vascular Inflammation, Oxidative Stress, and Endothelial Injury. PLoS One. 2016; 11: e0166790 doi: 10.1371/journal.pone.0166790 27861612 10.1371/journal.pone.0166790 PMC5115793

[32] Zheng XT, Wu ZH, Wei Y, Dai JJ, Yu GF, Yuan F, et al Induction of autophagy by salidroside through the AMPK-mTOR pathway protects vascular endothelial cells from oxidative stress-induced apoptosis. Mol Cell Biochem. 2017; 425: 125–138. doi: 10.1007/s11010-016-2868-x 27848074 10.1007/s11010-016-2868-x

[33] Cao YJ, Zhang YM, Qi JP, Liu R, Zhang H, He LC. Ferulic acid inhibits H2O2-induced oxidative stress and inflammation in rat vascular smooth muscle cells via inhibition of the NADPH oxidase and NF-κB pathway. Int Immunopharmacol. 2015; 28: 1018–1025. doi: 10.1016/j.intimp.2015.07.037 26330101 10.1016/j.intimp.2015.07.037

[34] Acker H. The oxygen sensing signal cascade under the influence of reactive oxygen species. Philos Trans R Soc Lond B Biol Sci. 2005; 360: 2201–2210. doi: 10.1098/rstb.2005.1760 16321790 10.1098/rstb.2005.1760 PMC1569600

[35] Lee SH, Gupta MK, Bang JB, Bae H, Sung HJ. Current progress in Reactive Oxygen Species (ROS)-Responsive materials for biomedical applications. Adv Healthc Mater. 2013; 2: 908–915. doi: 10.1002/adhm.201200423 25136729 10.1002/adhm.201200423 PMC4146500

[36] Ling X, Zhang S, Shao P, Wang P, Ma X, Bai M. Synthesis of a reactive oxygen species responsive heterobifunctional thioketal linker. Tetrahedron Lett. 2015; 56: 5242–5244. doi: 10.1016/j.tetlet.2015.07.059 26309336 10.1016/j.tetlet.2015.07.059 PMC4545510

[37] Wilson DS, Dalmasso G, Wang L, Sitaraman SV, Merlin D, Murthy N. Orally delivered thioketal nanoparticles loaded with TNF-α-siRNA target inflammation and inhibit gene expression in the intestines. Nat Mater. 2010; 9: 923–928. doi: 10.1038/nmat2859 20935658 10.1038/nmat2859 PMC3142359

